# Post-stroke headache: a review of epidemiology, pathophysiology, and clinical management

**DOI:** 10.3389/fnins.2026.1766866

**Published:** 2026-03-18

**Authors:** Dabao Yao, Luwei Nie, Danyang Chen, Shiling Chen, Xuan Wu, Chao Pan, Yingxin Tang, Na Liu, Zhouping Tang

**Affiliations:** Department of Neurology, Tongji Hospital, Tongji Medical College, Huazhong University of Science and Technology, Wuhan, Hubei, China

**Keywords:** cerebrovascular disease, ICHD-3 classification, post-stroke headache, stroke mimics, therapeutic strategies

## Abstract

Post-stroke headache (PSH) and its chronic counterpart, persistent post-stroke headache (PPSH), represent significant but frequently overlooked complications of cerebrovascular disease that adversely affect rehabilitation and quality of life. This review provides an updated synthesis of PSH, following its formal classification in the International Classification of Headache Disorders, 3rd edition (ICHD-3). We examine the epidemiology of PSH, noting a prevalence range of 6–44% in ischemic stroke survivors, with risk factors including younger age, female sex, and posterior circulation lesions. The pathophysiology is explored as a complex interplay involving the trigeminovascular system, neurogenic inflammation, and central sensitization, often exacerbated by structural factors such as edema and stroke topography. Clinical phenotypes vary, predominantly presenting as tension-type, though migraine-like features occur. Furthermore, this review highlights the critical role of headache as a sentinel symptom in the differential diagnosis of distinct stroke etiologies such as cervical artery dissection, reversible cerebral vasoconstriction syndrome, and cerebral venous thrombosis. A major finding is the significant gap in evidence-based management; current therapeutic strategies often rely on extrapolating data from primary headache disorders, with unverified safety profiles for newer agents such as triptans and calcitonin gene-related peptide (CGRP) antagonists in the post-stroke population. We conclude by emphasizing the urgent need for randomized controlled trials to establish safe, effective pharmacological and non-pharmacological interventions for this disabling condition.

## 1 Introduction

Among the many disabling sequelae of stroke, headache is a common but frequently overlooked complication that can significantly impair rehabilitation and quality of life (Carey et al., 2022). While motor and cognitive deficits are well-recognized, pain syndromes—specifically post-stroke headache (PSH) and persistent post-stroke headache (PPSH)—often remain underdiagnosed and undertreated. This represents a significant gap in comprehensive post-stroke care, as these conditions not only cause physical suffering but also contribute to psychological distress, further impairing rehabilitation efforts (Delpont et al., 2018; Harrison and Field, 2015).

Headache can manifest either as an acute symptom at the onset of a cerebrovascular event or as a persistent, chronic problem that endures long after the initial injury. It is crucial to distinguish between the acute headache that occurs in close temporal relation to a stroke and the chronic form, known as PPSH, which is the primary focus of this review. Observational studies report that acute headache occurs in close temporal relation to an acute ischemic stroke in 15–40% of patients (Diener et al., 2008). Reported prevalence varies widely due to heterogeneity in diagnostic criteria, timing of assessment, and inclusion of patients with preexisting headache disorders. A prospective study of 240 patients found headache occurred in 38% of all acute stroke cases, with a significantly higher prevalence in hemorrhagic (64.5%) versus ischemic stroke (32%) (Arboix et al., 1994). Despite its frequency, headache attributed to ischemic stroke or transient ischemic attack (TIA) is often underdiagnosed. This lack of recognition can be detrimental, as headache accompanying an ischemic stroke may be an independent predictor of neurological deterioration (Xie et al., 2022). The presence of PSH is associated with a significant disability burden, contributing to comorbid depression, cognitive dysfunction, and a general impairment of quality of life (Rost et al., 2022). For many patients, the chronic pain becomes a primary obstacle to participating in rehabilitation and resuming daily activities. The failure to adequately address this symptom represents a significant gap in comprehensive post-stroke care.

A pivotal moment in the recognition of chronic headache as a distinct post-stroke complication was the publication of the International Classification of Headache Disorders, 3rd edition (ICHD-3) in 2018. This edition was the first diagnostic manual to formally include criteria for PPSH (Arnold, 2018). The ICHD-3 defines PPSH as a new headache that develops in close temporal relation to an ischemic stroke and persists for more than 3 months. This formal classification distinguishes the chronic form of PSH from the acute headache that may occur at the time of the cerebrovascular event. This diagnostic framework is essential for standardizing research and improving clinical recognition, moving PPSH from an overlooked symptom to a recognized clinical entity deserving of dedicated attention.

While headache is an important symptom in a wide range of cerebrovascular diseases, from ischemic and hemorrhagic stroke to cervical artery dissection (CeAD) and cerebral venous thrombosis (CVT), the characteristics and management of the persistent headache that follows a stroke are not well defined (Rothrock and Diener, 2021; Lu et al., 2020). Despite its prevalence and the significant disability it confers, there remains a paucity of knowledge regarding its specific characteristics, underlying pathophysiology, and optimal management strategies. There are currently no evidence-based guidelines to direct the treatment of PPSH, forcing clinicians to extrapolate from therapies for primary headache disorders, a practice that may not be optimal or safe in the post-stroke population (Plecash et al., 2019). This review aims to provide a comprehensive and updated overview of PSH, with a particular focus on the persistent form (PPSH). We will discuss its epidemiology, clinical presentation, and hypothesized pathophysiological mechanisms. Furthermore, we will explore the critical role of headache as a sentinel symptom in the differential diagnosis of various stroke etiologies. The diagnostic workup, current management approaches, including the safety of specific migraine therapies, and non-pharmacological options will be examined in detail. By synthesizing the available literature, this review seeks to outline the current state of knowledge, identify critical evidence gaps, and propose future research priorities for this disabling condition.

### 1.1 Methods and literature search strategy

This article is a narrative review focusing on the epidemiology, clinical characteristics, pathophysiology, and management of headache associated with cerebrovascular disease. We conducted a comprehensive literature search using the PubMed, EMBASE, and Cochrane Library databases for articles published from inception up to November 5th, 2025. The search strategy employed a combination of medical subject headings (MeSH) and free-text terms including: “headache,” “stroke,” “ischemic stroke,” “hemorrhagic stroke,” “cerebrovascular disease,” “post-stroke headache,” “sentinel headache,” and “pathophysiology.” We prioritized systematic reviews, meta-analyses, randomized controlled trials, and large prospective observational studies. Studies were restricted to those published in the English language. Articles were selected based on their relevance to the core themes of epidemiology, clinical phenotyping, pathophysiological mechanisms, and differential diagnosis. Reference lists of identified key articles were also manually reviewed to identify additional relevant sources.

## 2 Epidemiology and classification of PSH

### 2.1 Prevalence and epidemiology

The prevalence of headache associated with stroke exhibits considerable variability across studies. To understand the true burden of this condition, it is necessary to differentiate between specific stroke etiologies and the temporal phase of the headache, while also accounting for significant methodological differences in the available literature.

In the context of acute ischemic stroke, the reported prevalence of new-onset headache ranges widely. A systematic review and meta-analysis of 50 studies identified a prevalence range of 6–44%, with a pooled estimate of 14% (Harriott et al., 2020). In China, a large-scale meta-analysis of 98 studies reported a slightly higher overall pooled prevalence of 18.9% (Xie et al., 2022). The prevalence is markedly higher in patients with hemorrhagic stroke due to the mechanisms of mass effect and direct meningeal irritation. A prospective clinical study explicitly compared these populations, finding that acute headache occurred in 64.5% of patients with hemorrhagic stroke compared to only 32% of those with ischemic stroke (Arboix et al., 1994). Similarly, headache is a frequent symptom in TIA, with estimates between 26 and 36% (Oliveira and Sampaio Rocha-Filho, 2019).

A distinct epidemiological category is the chronification of acute symptoms into persistent post-stroke headache (PPSH). Defined as headache persisting for more than 3 months after the vascular event, PPSH is estimated to affect between 1 and 23% of stroke survivors (Lai et al., 2018; Chan and Thaler, 2023). These persistent headaches often manifest with tension-type features and can present with greater severity than the initial acute symptoms (Lai et al., 2018).

It is critical to note that methodological heterogeneity heavily influences these varying estimates. Timing of assessment is a fundamental factor; studies focusing on the acute phase capture immediate vascular responses, whereas estimates at 3 months reflect persistent phenotypes. Furthermore, stroke severity introduces profound selection bias, as the systematic exclusion of patients with aphasia leads to a consistent underestimation of incidence. Additionally, the assessment tool utilized dictates reporting rates; prospective interviews yield significantly higher prevalence rates compared to retrospective registry reviews, which often miss headaches that were not the primary reason for hospital admission (Harriott et al., 2020). A summary of these prevalence estimates, stratified by study population and outcome, is provided in Table 1.

**Table 1:** Summary of prevalence estimates for post-stroke headache by population and outcome.

Study/source	Study design	Population/subtype	Diagnostic method	Outcome measure	Prevalence estimate
Harriott et al. (2020)	Systematic review and meta-analysis (50 studies)	Ischemic stroke (global)	Mixed (retrospective and prospective)	Acute headache attributed to stroke	Range: 6–44% (pooled: 14%)
Xie et al. (2022)	Systematic review and meta-analysis (98 studies)	Ischemic stroke (China)	Mixed	Acute headache attributed to stroke	Pooled: 18.9% (rural: 24.9%)
Arboix et al. (1994)	Prospective clinical study (*n =* 240)	Ischemic stroke (*n =* 195) Hemorrhagic stroke (*n =* 45)	Direct interview (acute phase)	Acute headache attributed to stroke / intracranial hemorrhage	Ischemic: 32% hemorrhagic: 64.5%
Oliveira et al. (2023)	Narrative review	Ischemic stroke TIA	Review of literature	Acute headache attributed to stroke / TIA	Stroke: 7.4–34% TIA: 26–36%
Lai et al. (2018) and Chan and Thaler (2023)	Narrative review	Ischemic and hemorrhagic stroke	Review of literature	PPSH (>3 months)	Range: 1–23%

### 2.2 Classification

The ICHD-3 operationalizes the diagnosis of stroke-related headaches by distinguishing between acute and persistent forms. It defines acute headache attributed to ischemic stroke or TIA as a headache that develops simultaneously with or in very close temporal relation to the onset of focal neurological deficits (Olesen, 2018). This headache is a direct consequence of the cerebrovascular event itself. The pain usually has a concomitant onset with the neurological symptoms and tends to improve over time as the acute phase of the stroke resolves (Oliveira and Sampaio Rocha-Filho, 2019). The characteristics of this acute headache can provide clues to the underlying stroke (e.g., occipital localization suggesting posterior circulation involvement, or ipsilateral neck pain suggesting CeAD), but are not specific enough for a definitive diagnosis without further investigation (Goddeau and Alhazzani, 2013; Salerno et al., 2022; Yaghi et al., 2024). Its presence is considered a common accompanying symptom of cerebrovascular disease (Lu et al., 2020).

A significant advancement in the ICHD-3 was the formal recognition of PPSH (Arnold, 2018). As defined in the ICHD-3, the diagnosis of PPSH is applied when the acute headache attributed to stroke persists for more than 3 months (Table 2). This distinction is crucial as it separates the acute, self-limiting headache from a chronic, disabling pain disorder that requires long-term management. While the formal recognition of PPSH is a significant advancement, applying these criteria in the post-stroke population has limitations. These include strict temporal thresholds that may not capture delayed onset pain, and challenges in assessing headache phenotypes in patients with post-stroke cognitive impairment or aphasia.

**Table 2:** Classification and definition of post-stroke headache entities based on ICHD-3 criteria.

Terminology	ICHD-3 code	Definition and criteria	Clinical context
Acute headache attributed to ischemic stroke	6.1.1	A new headache developing simultaneously with or in close temporal relation to signs of ischemic stroke.	Occurs at the onset of the cerebrovascular event. Typically resolves as the stroke stabilizes.
Persistent headache attributed to past ischemic stroke (PPSH)	6.1.1.2	A headache caused by ischemic stroke that persists for >3 months after stabilization of the stroke.	Represents the chronification of the acute headache. Often resembles tension-type headache.
Acute headache attributed to non-traumatic intracranial hemorrhage	6.2	A new headache developing in close temporal relation to nontraumatic intracranial hemorrhage.	Typically more severe (“thunderclap”) than ischemic stroke headache. Strongly associated with mass effect.
Post-stroke headache (PSH)	N/A*	An umbrella term used in clinical literature to encompass both acute and persistent forms.	Used generally when discussing the broad phenomenon or when the timeframe is unspecified.

*PSH is a descriptive clinical term, whereas ICHD-3 codes refer to specific diagnostic entities. ICHD-3, International Classification of Headache Disorders, 3rd edition.

## 3 Clinical characteristics and phenotypes of PSH

### 3.1 Temporal profile

The temporal relationship between headache and a cerebrovascular event is a key diagnostic feature. For an acute headache to be attributed to a stroke or TIA, its onset is usually concomitant with the focal neurological deficits. In a prospective study, the headache associated with ischemic stroke had a mean duration of approximately 25 h, which was significantly shorter than the headache seen in hemorrhagic stroke, which lasted a mean of 64.5 h (Arboix et al., 1994). This acute headache typically improves over time.

However, a subset of patients experiences a transition from this acute symptom to a chronic pain disorder. This process of chronification leads to the diagnosis of PPSH. The headaches in PPSH may evolve to become more frequent and severe than the acute headaches experienced at stroke onset (Lebedeva et al., 2022). The development of this chronic form represents a challenge for long-term management, as it transforms a transient symptom into a long-term source of disability that impacts recovery and quality of life. Understanding the factors that drive this chronification is one of the research areas for future investigation.

### 3.2 Clinical phenotype and pain characteristics

The clinical presentation of PSH is diverse, yet distinct phenotypic patterns allow for characterization. The most frequently observed phenotype resembles a tension-type headache (Lebedeva et al., 2022). Clinically, this presents as mild to moderate pain that is typically bilateral and pressing or tightening in quality. Crucially, this phenotype lacks the ancillary symptoms often seen in primary neurovascular headaches: nausea, vomiting, photophobia, and phonophobia are generally absent. Headache manifestations in ischemic stroke are variable. However, a significant minority of patients present with a migraine-like phenotype (Angus-Leppan, 2013). In these instances, the headache may be unilateral, pulsating, and accompanied by nausea or sensitivity to light and sound. This presentation is more common in patients with a pre-existing history of migraine, suggesting a “primed” neurovascular system (Oliveira et al., 2023; Coban et al., 2016). The distinction can be clinically challenging and requires distinguishing between an acute migraine attack mimicking stroke (e.g., hemiplegic migraine) and a true ischemic stroke triggering a secondary migraine-like headache. Furthermore, the migraine-like phenotype may point toward specific underlying genetic arteriopathies that predispose to both stroke and headache, such as CADASIL (cerebral autosomal dominant arteriopathy with subcortical infarcts and leukoencephalopathy) and MELAS (mitochondrial myopathy, encephalopathy, lactic acidosis and stroke-like episodes) (Kopishinskaya and Gustov, 2015).

Acute pain characteristics—specifically location and severity—are often correlated with the stroke mechanism and topography, though the extent to which these patterns persist into the chronic PPSH phase remains under investigation. In ischemic stroke, the headache is frequently focal (reported in 74% of cases) and of moderate intensity (Arboix et al., 1994). In sharp contrast, the headache associated with hemorrhagic stroke is more often diffuse (52%) and is described as severe or incapacitating in up to 70% of patients (Arboix et al., 1994). The vascular territory involved is also predictive; strokes occurring in the posterior circulation (vertebrobasilar system) are more strongly associated with headache than those in the anterior (carotid) circulation. This is likely due to the dense innervation of the large arteries of the posterior fossa by the upper cervical roots and the trigeminal nerve, making them more sensitive to the mechanical and chemical distortions of a stroke (Kumral et al., 1995).

## 4 Pathophysiology of headache in cerebrovascular disease

While the initial vascular injury triggers acute pain, the following mechanisms explain how this nociception may evolve into the central sensitization and chronic neuroinflammation characteristic of PPSH.

### 4.1 Role of stroke topography

The location of the ischemic lesion within the brain is a critical determinant of the likelihood and characteristics of PSH. A consistent finding across multiple studies is that strokes occurring in the posterior circulation are more strongly associated with headache than those in the anterior circulation (Kumral et al., 1995). This predilection for the posterior circulation may be related to the denser innervation of posterior circulation vessels by nociceptive afferents from the trigeminal system.

The depth of the lesion also plays a significant role. Cortical infarcts are more frequently associated with headache than subcortical or lacunar infarcts (Oliveira and Sampaio Rocha-Filho, 2019). One study found that 56.5% of patients with cortical strokes experienced headache, in contrast to only 26.5% of those with subcortical strokes (Arboix et al., 1994). This suggests that irritation of the richly innervated pial and cortical arteries is a key mechanism. Lacunar infarctions, which occur in deep brain structures, are less likely to cause headache.

Specific brain structures are also implicated. Midbrain lesions have been identified as one of the major risk factors for developing ischemic stroke-related headaches (Wijeratne et al., 2023). The midbrain is a crucial hub for pain processing pathways, and damage to this area could disrupt normal nociceptive modulation. Similarly, cerebellar strokes are also known to be associated with headache (Altiparmak et al., 2021). The consistent link between stroke topography and headache risk points to the direct involvement of neurovascular structures and central pain pathways in the generation of post-stroke pain.

Beyond the location of the primary infarct, the underlying state of the cerebral microvasculature may also be a predisposing factor. While direct evidence is still needed, it is plausible that a significant burden of cerebral small vessel disease, often visualized as white matter hyperintensities on magnetic resonance imaging (MRI), could contribute to the risk of developing PSH and its chronification (Sargurupremraj et al., 2020; Zhang et al., 2024). Small vessel disease reflects a state of chronic endothelial dysfunction and impaired vasoreactivity. This underlying vasculopathy could lower the threshold for trigeminovascular activation or impair the resolution of neurogenic inflammation following an acute ischemic event (Wardlaw et al., 2019; Sun et al., 2025). Future prospective studies should aim to quantify small vessel disease burden and investigate its role as a potential predictor for the development and persistence of PSH.

### 4.2 Activation of the trigeminovascular system and neurogenic inflammation

The trigeminovascular system is widely considered a cornerstone in the pathophysiology of many headache types, including those secondary to cerebrovascular disease (Wang W. et al., 2024). This system consists of nociceptive neurons originating in the trigeminal ganglion that innervate the large cerebral arteries, pial arteries, and dura mater. Activation of these perivascular afferents is thought to be a primary mechanism for headache pain generation (Boinpally et al., 2024). In the context of an ischemic stroke, several triggers can activate this system. The ischemic event itself, along with subsequent reperfusion, can lead to the release of various inflammatory mediators and ions that sensitize and activate these trigeminal nerve endings (Levin et al., 2022).

**Figure 1:**
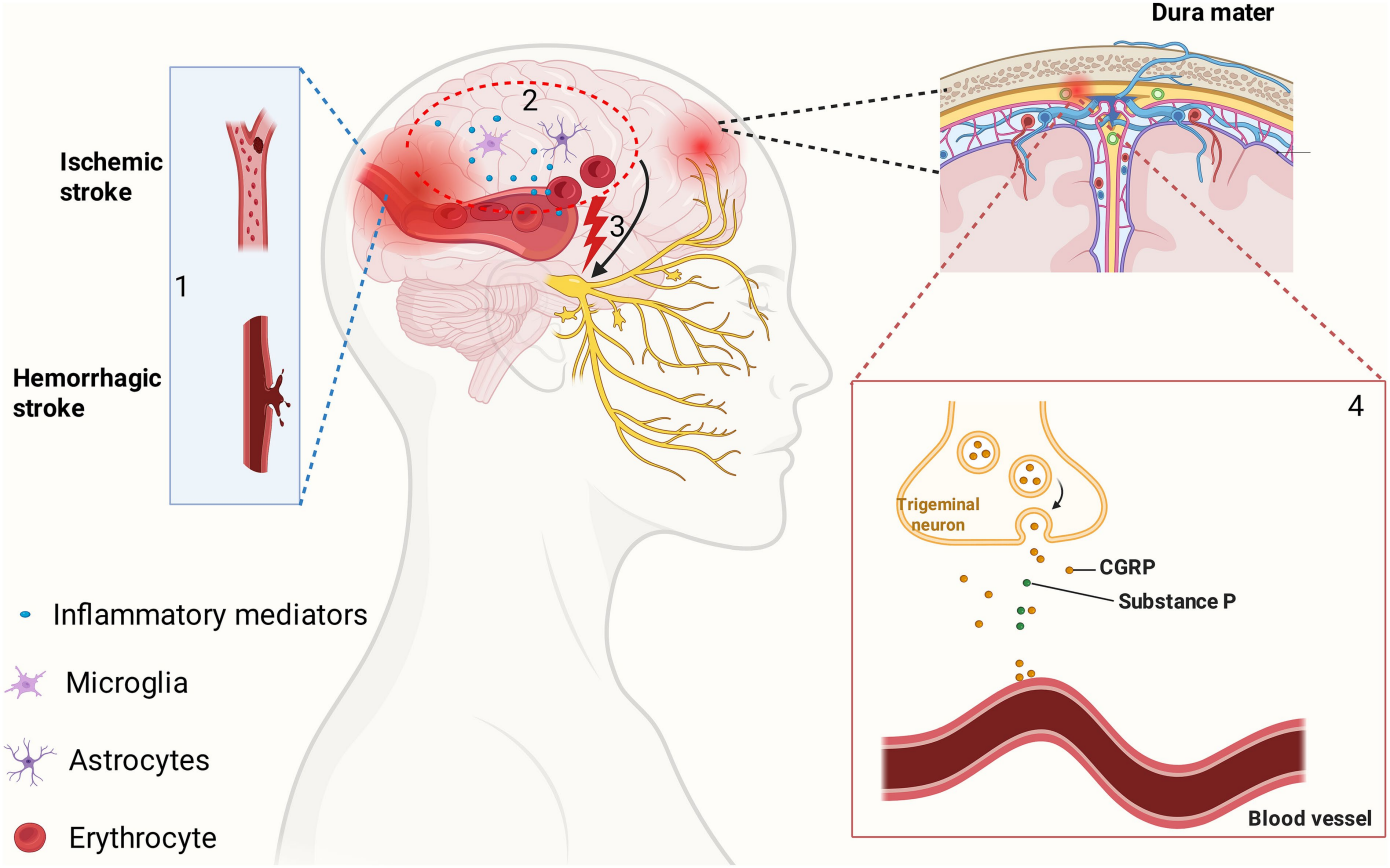
The pathophysiological cascade of post-stroke headache. (1) A stroke occurs, either ischemic (occlusion) or hemorrhagic (rupture). (2) The parenchymal injury and disruption of the blood–brain barrier trigger a local neuroinflammatory response, involving the activation of glial cells (microglia, astrocytes) and the release of inflammatory mediators and, in the case of hemorrhage, blood products (erythrocytes). (3) These substances sensitize and activate perivascular nociceptive afferents of the trigeminal nerve, generating a pain signal. This signal travels orthodromically to the brainstem to be perceived as pain. (4) The activation also causes an antidromic signal, leading to the release of vasoactive neuropeptides, principally CGRP and substance P, from trigeminal nerve endings onto dural and pial blood vessels. This results in vasodilation and plasma protein extravasation (neurogenic inflammation), further sensitizing nociceptors and perpetuating the headache. CGRP, calcitonin gene-related peptide. Figure created with BioRender.com

This activation can, in turn, trigger a process known as neurogenic inflammation. Activated trigeminal fibers release neuropeptides such as calcitonin gene-related peptide (CGRP), substance P, and neurokinin A into the perivascular space. These substances cause vasodilation and increase plasma protein extravasation from dural blood vessels, leading to a sterile inflammatory response that further sensitizes nociceptors and perpetuates the pain signal (Baser et al., 2020) (Figure 1). This mechanism is well-established in primary headaches like migraine and is believed to play a role in PSH as well (Mohanan et al., 2023). The pathophysiology of headache in cerebral hyperperfusion syndrome, a complication following revascularization procedures, is also thought to involve impaired autoregulation and disruption of the blood–brain barrier, which would facilitate the processes of neurogenic inflammation. The higher incidence of headache in posterior circulation strokes might be explained by a greater density of trigeminal innervation in the vertebrobasilar system, making it more susceptible to this type of activation.

### 4.3 Mechanisms of central pain and sensitization

While peripheral activation of the trigeminovascular system explains the initial nociceptive signal, the development of PPSH likely involves changes within the central nervous system (CNS), a process known as central sensitization (Zhou et al., 2022). Central sensitization refers to a state of hyperexcitability in the central pain-processing neurons, where the system becomes more responsive to both noxious and non-noxious stimuli. This can lead to allodynia (pain from a stimulus that does not normally provoke pain) and hyperalgesia (an increased response to a painful stimulus).

In the context of stroke, the vascular lesion itself can directly damage central pain pathways, particularly those involving the thalamus and brainstem (Castel et al., 2013). Thalamic pain syndrome, a form of central post-stroke pain (CPSP), is a classic example of pain arising from a lesion in central sensory structures (Hayashi et al., 2007). Damage to these areas can lead to a disinhibition or spontaneous firing of nociceptive neurons, generating pain in the absence of ongoing peripheral input. Functional neuroimaging studies using techniques such as functional MRI have contributed to understanding these central mechanisms by allowing researchers to map the neural systems involved in headache pathogenesis and brain reorganization after stroke (Weiller et al., 2010; Mechtler, 2004). The persistence of headache long after the acute ischemic event has resolved strongly suggests that these central mechanisms, rather than ongoing peripheral triggers, are responsible for maintaining the chronic pain state in PPSH (Kang et al., 2025).

### 4.4 Role of mass effect, edema, and hemorrhagic transformation

In the acute phase of a stroke, mechanical factors can contribute significantly to the generation of headache. Large ischemic strokes can lead to cytotoxic and vasogenic edema, causing swelling of the affected brain tissue (Diener et al., 2008; Balami et al., 2011). This brain edema can increase intracranial pressure (ICP) and exert a mass effect, physically stretching or compressing pain-sensitive intracranial structures like the dura mater, blood vessels, and cranial nerves (del Sanchez Rio and Moskowitz, 1999). This mechanical stimulation of nociceptors is a potent trigger for headache. The headache associated with CVT is also largely attributed to increased ICP (Nascimento et al., 2016).

Hemorrhagic transformation of an ischemic infarct, where bleeding occurs into the area of dead tissue, is another important neurological complication that can cause or worsen headache (Diener et al., 2008; Balami et al., 2011). The extravasated blood and its breakdown products are highly irritating to the meninges and perivascular nerves, which provokes a robust inflammatory response and nociceptor activation. This explains why headache is significantly more common and severe in hemorrhagic strokes compared to ischemic strokes (Rothrock, 2004). Similarly, the severe headache in aneurysmal subarachnoid hemorrhage (SAH) is a direct result of blood filling the subarachnoid space (Sorrentino et al., 2022). Conditions like cerebral hyperperfusion syndrome can also lead to intracerebral hemorrhage, manifesting with severe headache, seizures, and focal deficits (Guo et al., 2023). Thus, mass effect, edema, and the presence of blood are powerful acute triggers for headache in the context of cerebrovascular events.

### 4.5 The inflammatory cascade

The inflammatory response is a central feature of both stroke and many systemic diseases that increase stroke risk, and it plays a crucial role in the pathophysiology of associated headaches. Following an ischemic event, the brain triggers a wave of inflammatory cytokines that can induce endothelial cell dysfunction and a prothrombotic state, increasing the risk of further thrombosis (Paolucci et al., 2021). This systemic inflammation can also breach the blood–brain barrier, allowing pro-inflammatory cytokines to enter the CNS or stimulating microglia and astrocytes to produce their own cytokines within the brain (Huang et al., 2021). The clinical implications of severe systemic inflammation driving both stroke and headache are discussed further in the context of specific infections like COVID-19 and tuberculous meningitis (see section 9).

## 5 Etiologies and differential diagnosis of headache in cerebrovascular disease

While the primary focus of this review is post-stroke sequelae, it is vital to recognize headache as a presenting symptom of specific stroke etiologies. Headache, particularly when acute and severe, can be the leading or presenting symptom of a range of serious cerebrovascular diseases that cause stroke (Rothrock and Diener, 2021). Its presence in conjunction with focal neurological deficits, or even as an isolated symptom, necessitates a high index of suspicion and a judicious diagnostic approach in the emergency setting. A structured evaluation is essential to distinguish between benign primary headache disorders and these secondary, life-threatening causes. The clinical association between headache and stroke is complex, and accurate diagnosis often relies on recognizing specific patterns and utilizing appropriate neuroimaging (Khan et al., 2025).

### 5.1 CeAD

CeAD, involving the carotid or vertebral arteries, is a frequent cause of stroke, especially in patients younger than 50 years of old (Grond-Ginsbach et al., 2018). Headache and/or neck pain are the most common immediate complications, occurring in 65–95% of cases (Keser et al., 2022). The pain is often a sentinel symptom, preceding the onset of ischemic events (Nagumo et al., 2003). In carotid artery dissection, the pain is typically unilateral head, facial, or neck pain. This may be associated with a partial Horner’s syndrome (miosis and ptosis), found in about 25% of CeAD cases, or pulsatile tinnitus (Keser et al., 2022; Sztajzel et al., 2017; Baumgartner and Bogousslavsky, 2005). In vertebral artery dissection (VAD), the characteristic presentation is pain in the posterior neck and occipital region of the head (Matsumoto et al., 2023; Arnold and Bousser, 2005). Dizziness or vertigo is also very common symptoms in VAD, reported in 58% of patients (Gottesman et al., 2012). While often spontaneous or following minor neck trauma, VAD can be triggered by less recognized events like violent coughing (Khalid et al., 2024). The ischemic stroke results from thromboembolism originating from the site of dissection or, less commonly, from hemodynamic compromise due to stenosis or occlusion (Wang et al., 2025). Given that symptoms like headache and neck pain are common in the emergency department, clinicians must maintain a high index of suspicion for CeAD to ensure timely diagnosis and initiation of antithrombotic therapy to prevent stroke.

### 5.2 Reversible cerebral vasoconstriction syndrome (RCVS)

RCVS is a critical differential diagnosis for acute severe headache, characterized by segmental, multifocal constriction of intracranial arteries that typically resolves within weeks (Nelson, 2024). The hallmark clinical feature is a recurrent “thunderclap” headache, a worst-ever headache that reaches maximum intensity within a minute (Erhart et al., 2023; Simonsen et al., 2025). While the initial brain imaging is often normal, about one-third to one-half of patients develop cerebrovascular complications, including convexity SAHs, lobar hemorrhages, and ischemic strokes, typically in arterial watershed territories. RCVS predominantly affects women, and one well-known manifestation is postpartum angiopathy. The condition has been described in numerous clinical settings and can overlap with posterior reversible encephalopathy syndrome and Takotsubo cardiomyopathy (Singhal, 2023). Diagnosis is supported by tools like the RCVS2 score, which helps differentiate it from mimics such as primary angiitis of the CNS. The pathophysiology remains largely unknown, but management involves removing vasoconstrictive triggers and symptomatic relief, often with calcium-channel blockers (Singhal, 2023). RCVS has also been reported in the pediatric population, where it is surprisingly more common in males and can be triggered by events like exercise or trauma (Fujii et al., 2021).

### 5.3 CVT

CVT is an uncommon but important cause of stroke, particularly in young and middle-aged individuals (Ding et al., 2024; Wang J. et al., 2024). Headache is the most frequent symptom of CVT, present in nearly 90% of patients, and it can sometimes be the only manifestation (Alamri et al., 2021). The headache associated with CVT has no single specific pattern; its onset can be acute or subacute over several days, it can be of any severity, and it is often diffuse but can be localized (Agostoni, 2004). This variability makes the diagnosis challenging, as it can mimic migraine, tension-type headache, or even SAH (Agostoni, 2004). The underlying mechanism is primarily an increase in ICP due to obstruction of venous outflow, which can lead to venous infarction, hemorrhage, and other symptoms like seizures, blurry vision, nausea, and altered consciousness (Sasidharan, 2012). Key risk factors include prothrombotic conditions, pregnancy (especially the puerperium), and oral contraceptive use (Wang et al., 2022). The wide spectrum of clinical presentations necessitates that clinicians consider CVT in the differential diagnosis of patients presenting with headache, stroke, or seizures, especially when risk factors are present (Oliveira et al., 2022). MRI and magnetic resonance venography are the diagnostic modalities of choice (Vogl et al., 1994).

### 5.4 Aneurysmal SAH

Aneurysmal SAH is a devastating type of hemorrhagic stroke that accounts for a small percentage of all strokes but contributes significantly to morbidity and mortality (Hao et al., 2023; Luo and Li, 2025). The classic presentation is a sudden, severe “thunderclap” headache, often described as the “worst headache of my life” (Patel et al., 2005). This headache is a direct result of arterial blood entering the subarachnoid space and irritating the meninges (Shea and Stahmer, 2012). While this presentation is classic, the clinical spectrum is wide, and misdiagnosis is common, especially in patients with a normal neurological exam and absent neck stiffness (Rice-Canetto et al., 2025). Headache is the most common chief complaint and can be an isolated finding. Other symptoms can include nausea, vomiting, photophobia, and altered mental status (Tate and Bushnell, 2011). A high index of suspicion in the emergency department is critical, as a missed diagnosis has devastating consequences. The diagnostic workup for a suspected SAH typically involves an immediate non-contrast head computed tomography (CT) scan, followed by a lumbar puncture (LP) to check for xanthochromia if the CT is negative (Gee et al., 2012). Once diagnosed, management focuses on securing the aneurysm to prevent rebleeding and managing complications like vasospasm and hydrocephalus (Demaerschalk, 2011).

### 5.5 Underlying arteriopathies

Several non-atherosclerotic, non-inflammatory arteriopathies can predispose individuals to stroke and present with headache. Fibromuscular dysplasia is an idiopathic, segmental arteriopathy of medium-sized arteries that classically affects young to middle-aged women (Narula et al., 2018). The extracranial carotid and vertebral arteries are commonly involved, and frequent symptoms include headaches and pulsatile tinnitus. More severe manifestations arise from complications such as CeAD and intracranial aneurysms, which can lead to ischemic stroke or SAH (Kesav et al., 2023).

Vasculitis, or inflammation of blood vessels, can affect the CNS and lead to stroke, particularly in children and young adults (Deb-Chatterji et al., 2021). CNS manifestations are diverse, ranging from headache and seizures to cerebrovascular accidents (Held et al., 2023). For instance, Lyme neuroborreliosis can cause cerebral vasculitis leading to ischemic stroke, often preceded by a prodromal stage with headaches (Akkurt et al., 2023). Antiphospholipid syndrome, or Hughes syndrome, is another condition that causes arterial and venous thrombosis and can present with a wide array of neurologic features, including stroke, migraine, and memory loss (Hughes, 2007; Sanna et al., 2005).

Cerebral amyloid angiopathy is a disorder of the elderly where amyloid peptides deposit in cerebral artery walls, leading to micro- and macro-hemorrhages (Han et al., 2015). A subset of patients develops cerebral amyloid angiopathy-related inflammation, a syndrome characterized by subacute neurobehavioral symptoms, headaches, seizures, and stroke-like signs, often with reversible T2 hyperintense lesions on MRI that respond to immunosuppression (Kirshner and Bradshaw, 2015). Other genetic arteriopathies like CADASIL are also associated with both migraine-like headaches and recurrent strokes (Walsh et al., 2000).

### 5.6 Stroke mimics

Differentiating an acute stroke from conditions that mimic its presentation is a common diagnostic challenge in emergency medicine, with misdiagnosis rates as high as 31% (Long and Koyfman, 2017). These “stroke mimics” can present with acute focal neurological deficits, making them difficult to distinguish from a true cerebrovascular event without careful evaluation and neuroimaging (El-Wahsh et al., 2021).

Hemiplegic migraine is a classic stroke mimic. As a rare variant of migraine with aura, it can cause transient, unilateral motor weakness, often accompanied by sensory and visual disturbances, which can be clinically indistinguishable from a TIA or stroke (Perrotta et al., 2018). The key is the careful history, which often reveals a personal or family history of similar episodes and a typical migrainous headache that follows the neurological deficits.

Seizures are another common mimic. The postictal state following a seizure can include a focal neurological deficit known as Todd’s paresis, which is a temporary weakness of a limb or one side of the body. This can closely resemble the hemiparesis of a stroke. The history of a witnessed seizure is crucial for diagnosis. Headache is also a common postictal symptom (Van Viet et al., 1990).

Functional neurological disorders, historically known as conversion disorders, can also present with sudden-onset weakness, sensory loss, or other deficits that mimic stroke (Macleod, 2022). The diagnosis often relies on the presence of positive clinical signs (e.g., Hoover’s sign) that are inconsistent with an organic neurological lesion. Other mimics include metabolic disturbances (e.g., hypoglycemia), systemic infections, space-occupying lesions like tumors or abscesses, and complex headaches (Finkelstein et al., 2021). While history and physical examination are key, any patient with a sudden-onset, objective, focal neurological deficit should be evaluated emergently as an acute stroke until proven otherwise, typically with neuroimaging.

The “Headache-Plus” concept serves as a critical clinical heuristic in the emergency evaluation of these patients (Tabatabai and Swadron, 2016). Under this framework, when an acute headache (the “Headache”) is accompanied by focal neurological signs (the “Plus”)—the probability of a secondary vascular etiology is significantly elevated. Given the time-sensitive nature of reperfusion therapies, such a clinical presentation warrants a high index of suspicion and should generally prioritize the activation of acute stroke protocols, even if the headache is the patient’s primary or most distressing complaint. This approach helps ensure that clinicians are not distracted by the intensity of the pain, thereby avoiding the common pitfall of prematurely diagnosing a primary headache disorder before a secondary cerebrovascular event has been appropriately excluded through neuroimaging.

## 6 Risk factors and associated comorbidities

### 6.1 Demographic and patient-related factors

Several patient-specific factors have been consistently identified as increasing the risk for developing headache after a stroke. Female sex is one of the most robustly reported risk factors. A meta-analysis found that female sex was associated with an odds ratio of 2.06 (95% confidence interval: 1.44–2.96) for developing ischemic stroke-related headache (Xie et al., 2022). Another systematic review calculated a pooled odds ratio of 1.25 for female sex, confirming that women have greater odds of experiencing headache associated with ischemic stroke (Harriott et al., 2020). This increased susceptibility is also noted in other related conditions; for instance, RCVS predominantly affects women, and fibromuscular dysplasia is also more common in females (Santos Neto et al., 2024).

Patients who develop PSH tend to be younger than those who do not (Ge et al., 2022). This may reflect different stroke etiologies in younger populations, such as CeAD, which is a common cause of stroke in those under 50 and frequently presents with headache (Keser et al., 2022).

Perhaps the strongest predictor is a pre-existing primary headache disorder, particularly migraine. A meta-analysis reported a history of headache as a major risk factor, with an odds ratio of 3.24 for developing a new headache after an ischemic stroke (Xie et al., 2022). Similarly, a prospective study found that a history of vascular or tension-type headache was significantly more common in the group of stroke patients who developed headache (40.5%) compared to those who did not (23.5%) (Arboix et al., 1994). This suggests that individuals with a pre-existing sensitive or primed nociceptive system may be more vulnerable to developing a secondary headache following a cerebrovascular insult.

### 6.2 Stroke-related factors

The characteristics of the stroke itself are powerful determinants of headache risk. As detailed in the pathophysiology section (4.1), stroke topography is a paramount factor, with lesion location in the posterior circulation, cortex, or midbrain significantly increasing the likelihood of PSH. The underlying stroke etiology is also critical; for instance, thrombotic infarcts are more commonly associated with headache than lacunar infarcts, and specific causes like CeAD or CVT carry their own high intrinsic risk of headache (Bobker and Safdieh, 2021; Arboix et al., 1993).

### 6.3 Associated comorbidities and clinical confounders

PPSH does not exist in a vacuum; it is often intertwined with other common and disabling post-stroke complications that act as both comorbidities and clinical confounders. Distinguishing PPSH from these factors is a significant challenge in clinical practice.

Foremost among these confounders is the presence of pre-existing primary headache disorders. As noted, a history of migraine is a strong predictor of PSH. However, the stress of a stroke event, hospitalization, and altered sleep schedules can frequently trigger an exacerbation of pre-existing migraine (Oliveira et al., 2023). Clinicians often face the difficulty of determining whether a patient is suffering from a true secondary headache attributed to the vascular injury (PSH) or a worsening of their primary headache disorder. This distinction is crucial, as it may influence the choice of preventive medication.

Furthermore, the risk of medication-overuse headache (MOH) represents a significant potential confounder in the stroke population. Stroke survivors often require multiple medications and may self-medicate with over-the-counter analgesics to manage persistent pain. The chronic use of simple analgesics, opioids, or triptans can paradoxically lead to an increase in headache frequency and severity (Ashina et al., 2023). MOH can mask the natural history of PSH and render preventive therapies ineffective. Clinicians must carefully monitor analgesic intake to prevent the superimposition of MOH on the post-stroke pain syndrome.

There is also a notable comorbidity between PPSH and neuropsychiatric conditions, specifically depression and fatigue (Zhou et al., 2023). Pain itself is associated with the presence of depression and cognitive dysfunction after stroke, creating a bidirectional vicious cycle where each condition can exacerbate the other, leading to a greater impairment in overall quality of life (Meyer-Frießem et al., 2021). Depression can lower the pain threshold, making headaches more refractory to treatment, while chronic pain is a known driver of reactive depression. The management of post-stroke pain is crucial because its effective treatment may, in turn, improve both function and these comorbid conditions. For example, high-frequency repetitive transcranial magnetic stimulation has been shown to be an effective intervention for post-stroke depression, although it may have headache as a side effect (Liu et al., 2019).

Sleep disorders, particularly insomnia, are also a frequent but underestimated problem in patients with central neurological disorders, including stroke. Insomnia after a stroke can be a direct consequence of the brain injury, or it can arise secondary to other factors like pain, depression, or medications (Mayer et al., 2011). The presence of chronic headache can significantly disrupt sleep patterns, and conversely, poor sleep can lower the pain threshold and worsen headache severity. This complex, bidirectional relationship highlights the importance of a comprehensive assessment that screens for these associated comorbidities. Addressing sleep disturbances, for example with cognitive behavioral therapy or melatonin, may be a crucial component of an effective management plan for a patient with PPSH (Mukherjee et al., 2024; Fayyazi et al., 2022). Other factors that may contribute to headache persistence and are often comorbid in stroke patients include obstructive sleep apnea and musculoskeletal imbalances resulting from motor deficits (Witkowska et al., 2024).

## 7 Diagnostic workup and evaluation

### 7.1 Clinical history and neurological examination

The clinical evaluation of PSH requires distinguishing expected chronic post-stroke symptoms from new, potentially life-threatening vascular complications. Rather than a general headache workup, the focus should be on identifying “red flags”—such as a sudden change in headache phenotype, thunderclap onset, or the emergence of new focal neurological deficits—that necessitate urgent investigation (Membrilla et al., 2024). This targeted approach is essential to rule out critical secondary etiologies particularly relevant in stroke survivors, including recurrent ischemia, CeAD, and CVT.

### 7.2 Neuroimaging

While non-contrast CT is the standard for excluding acute hemorrhage, evaluating PSH often requires advanced vascular imaging. CT angiography or magnetic resonance angiography are necessary to visualize vessel patency and identify specific etiologies such as arterial dissection, aneurysm, or RCVS (Li et al., 2023). In cases where CVT is suspected, magnetic resonance venography is the imaging modality of choice (Sadik et al., 2022). For chronic PPSH, MRI is preferred over CT to assess for structural changes or subtle inflammatory pathologies that may contribute to central sensitization.

### 7.3 Laboratory and cerebrospinal fluid analysis

Laboratory evaluation serves as an adjunct in specific clinical scenarios. Markers of systemic inflammation (e.g., erythrocyte sedimentation rate, C-reactive protein) are essential when giant cell arteritis is suspected in older adults (Loder and Cardona, 2011). LP remains critical if subarachnoid hemorrhage is suspected despite normal imaging, or to exclude infectious and inflammatory conditions of the CNS that may mimic stroke presentations (Ditta et al., 2013; Topakian et al., 2008).

## 8 Management and treatment of PSH

**Figure 2:**
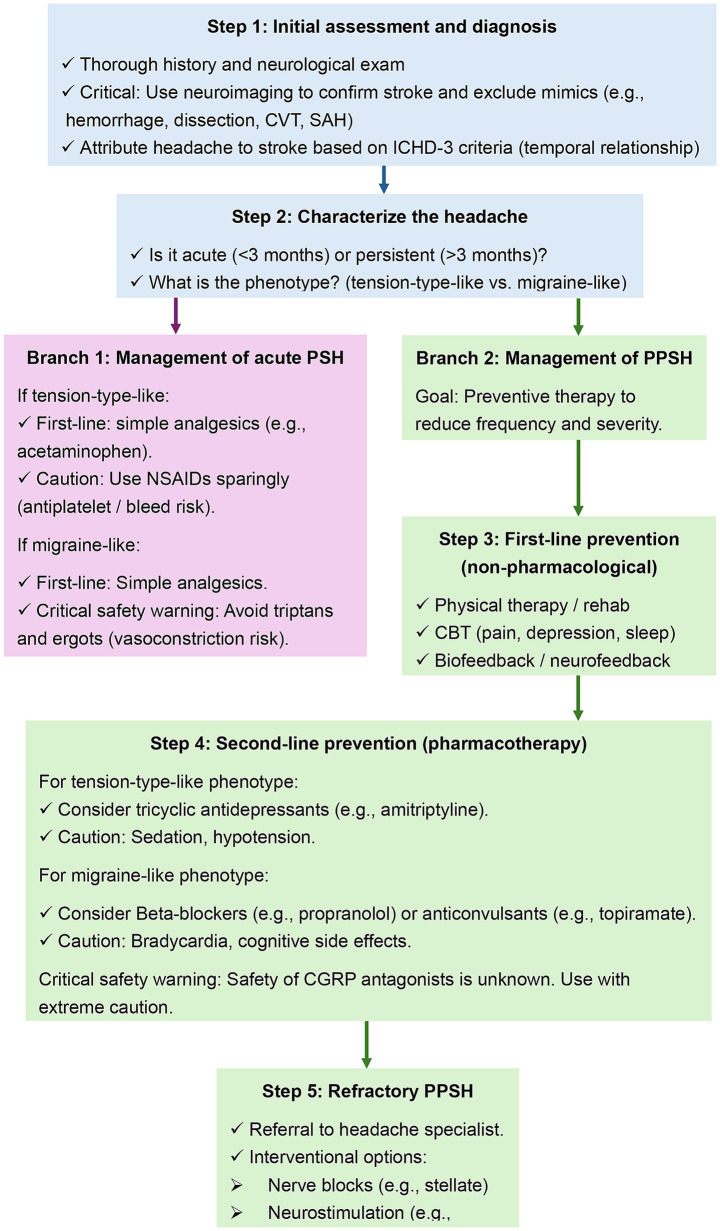
Proposed management algorithm for post-stroke headache. CVT, cerebral venous thrombosis; SAH, subarachnoid hemorrhage; PSH, post-stroke headache; NSAIDs, non-steroidal anti-inflammatory drugs; PPSH, persistent post-stroke headache; CBT, cognitive behavioral therapy; CGRP, calcitonin gene-related peptide.

The management of PSH is complex and lacks a strong evidence base. Therefore, a pragmatic, stepwise approach is necessary, as outlined in the proposed treatment algorithm (Figure 2). This framework guides the clinician from initial assessment through acute and preventive strategies, highlighting key safety considerations unique to the post-stroke population.

### 8.1 Acute symptomatic treatment

The management of acute headache in the setting of a cerebrovascular event is often challenging and guided more by clinical experience than by robust evidence from clinical trials. The choice of treatment depends heavily on the headache phenotype, the underlying stroke etiology, and patient comorbidities. For headaches that present with tension-type features, which is the most common phenotype, simple analgesics are often the first-line approach (Singhal, 2023).

However, the efficacy of these agents specifically for PSH has not been systematically studied. In some cases, the acute headache is a direct consequence of a specific pathology that requires targeted treatment. For example, the headache of cerebral hyperperfusion syndrome or hypertensive encephalopathy is primarily managed with strict blood pressure control (Bouri et al., 2011; Kirchoff-Torres and Bakradze, 2018). The headache of CVT may respond to measures that lower ICP (Neil et al., 2016).

The management of acute headache accompanying ischemic stroke is particularly complex due to concerns about medication safety. While non-steroidal anti-inflammatory drugs are often effective for acute pain, their use in stroke survivors warrants caution, particularly in patients on concurrent antiplatelet or anticoagulant therapy, given the potential for increased systemic or intracranial bleeding complications (Penner et al., 2022). A Cochrane review assessing nitric oxide donors (specifically glyceryl trinitrate) for acute stroke found that while glyceryl trinitrate lowered blood pressure, it did not improve clinical outcomes and significantly increased the incidence of headache as a side effect (Bath et al., 2017). The management of headache related to antiplatelet therapy for secondary stroke prevention also requires consideration; aspirin is associated with dose-dependent bleeding risk, and extended-release dipyridamole is known to cause headache, which may be managed by dose titration (Guthrie, 2011). Ultimately, there are no randomized trial data to guide the acute symptomatic treatment of PSH, representing a significant evidence gap.

### 8.2 Preventive pharmacotherapy for PSH

The management of PSH is even less defined than that of acute headache, with a notable lack of evidence-based guidelines. In the absence of specific trials for PSH, current practice relies on extrapolating treatment strategies from those used for primary headache disorders, such as chronic tension-type headache and chronic migraine. The choice of preventive medication is often guided by the headache’s clinical phenotype and the patient’s comorbidity profile.

Commonly used preventive agents for chronic daily headaches include tricyclic antidepressants (e.g., amitriptyline, nortriptyline), which may be particularly beneficial in patients with comorbid depression or insomnia, both common after stroke (Khammissa et al., 2020). Other options include anticonvulsants such as topiramate or gabapentin, and beta-blockers like propranolol. However, the use of these medications in the post-stroke population requires careful consideration of potential side effects, which can be particularly detrimental to the recovery process. For instance, the sedative and anticholinergic properties of tricyclic antidepressants like amitriptyline can cause daytime somnolence and cognitive dulling, directly impeding a patient’s ability to participate actively in physical, occupational, and speech therapy (Gonzalez-Martinez et al., 2022). Furthermore, the risk of orthostatic hypotension, a well-known effect of amitriptyline, is magnified in stroke survivors who may already have impaired autonomic function and motor deficits, significantly increasing the risk of falls and subsequent injury (Watanabe et al., 2022; Liang et al., 2024). Anticonvulsants such as topiramate can also cause cognitive slowing and word-finding difficulties, which can be difficult to distinguish from post-stroke cognitive deficits (Jiang et al., 2025). The potential for these specific adverse events complicates medication selection and underscores the need for therapies with better-proven safety profiles in this unique population. There are no clinical trials specifically evaluating the efficacy or safety of these preventive medications for PPSH. The need for methodologically sound research, including randomized controlled trials (RCTs), is paramount to establish effective and safe preventive therapies for this disabling condition.

### 8.3 Triptans and CGRP antagonists

The use of migraine-specific acute therapies, such as triptans and CGRP antagonists, in patients with PSH raises significant safety concerns, primarily due to their vascular mechanisms of action (Wilms et al., 2025). Triptans are serotonin (5-HT1B/1D) receptor agonists that cause vasoconstriction. Given that cerebrovascular disease is a contraindication for their use in patients with primary migraine, there is a theoretical risk that they could induce vasoconstriction in already compromised cerebral arteries, potentially worsening ischemia or causing a new vascular event in a post-stroke patient (Shapiro et al., 2019).

The newer CGRP antagonists, both monoclonal antibodies for prevention and small-molecule “gepants” for acute treatment, represent a potentially safer alternative. Their mechanism does not involve direct vasoconstriction (Diener et al., 2021). However, CGRP is a potent vasodilator that may play a protective role in the setting of cerebral ischemia. Therefore, blocking its effects could carry a theoretical risk of impairing collateral blood flow during ischemic events or hindering vascular recovery in the post-stroke population. Currently, there is no specific data on the safety or efficacy of either triptans or CGRP antagonists in the treatment of PPSH (Plecash et al., 2019). Their use should be approached with extreme caution, and likely avoided, until dedicated safety studies are conducted in this high-risk population. This highlights a critical area where research is needed to determine if these effective migraine treatments can be safely utilized by stroke survivors who develop migraine-like headaches.

### 8.4 Management of headache in specific stroke etiologies

The management of headache is often intrinsically linked to the treatment of the underlying cause of the stroke. In many cases, addressing the primary pathology leads to resolution of the associated headache. For CeAD, the mainstay of treatment is stroke prevention with antithrombotic therapy (either anticoagulation or antiplatelet agents) (Loufopoulos et al., 2025). While this is aimed at preventing thromboembolic events, the healing of the dissection itself, which occurs in many patients, often leads to the resolution of the initial head and neck pain. In rare cases of dissection with ongoing ischemic symptoms despite medical therapy, endovascular stenting or angioplasty may be considered (Ma et al., 2013).

In RCVS, management focuses on removing any identified triggers (e.g., vasoactive substances) and providing symptomatic relief for the severe headaches with analgesics. Oral calcium-channel blockers are commonly used, though evidence of their efficacy is not definitive (Singhal, 2023). For CVT, treatment typically involves systemic anticoagulation to prevent thrombus propagation and facilitate recanalization (Shrestha et al., 2016). In severe cases unresponsive to medical management, endovascular thrombolysis may be employed (Ye et al., 2022). The headache in CVT, often due to increased ICP, may improve as venous outflow is restored. For aneurysmal SAH, the priority is urgent surgical clipping or endovascular coiling of the aneurysm to prevent catastrophic rebleeding (Korkmaz Dilmen and Bonhomme, 2023). In each of these scenarios, the specific treatment of the vascular pathology is the effective way to manage the associated headache.

### 8.5 Non-pharmacological and interventional approaches

Given the limitations and potential side effects of pharmacotherapy, non-pharmacological and interventional approaches are gaining interest for the management of chronic post-stroke pain, including headache (Asadauskas et al., 2024). A multimodal treatment strategy that incorporates lifestyle interventions and conventional therapies may be a reasonable approach to address the complex interplay of clinical and psychosocial factors contributing to post-stroke pain.

Neurostimulation techniques have shown promise. High-frequency repetitive transcranial magnetic stimulation, while primarily studied for post-stroke depression, is an example of a neuromodulatory approach that could potentially be adapted for pain (Liu et al., 2019). Evidence for biobehavioral and interventional treatments in PPSH remains preliminary. For example, neurofeedback has been explored as a potential adjunctive therapy; however, data are currently limited to small studies and case series, and the requirement for extensive sessions (30-60) limits its feasibility (Kubik and Biedroń, 2013). Similarly, while case reports describe successful management of refractory thalamic pain using stellate ganglion blocks, these represent niche interventions lacking validation from large randomized controlled trials (Singh and Rajarathinam, 2024; Wilkinson et al., 2024).

Finally, rehabilitation and physical therapy play a crucial role. Musculoskeletal imbalances, spasticity, and contractures resulting from the stroke can themselves be sources of pain or contribute to the persistence of headache. A comprehensive rehabilitation program that addresses these issues can be an essential part of managing a stroke survivor’s overall pain burden.

## 9 Headache in special populations

Specific clinical contexts present unique challenges where headache acts as a critical sentinel symptom for cerebrovascular events. In pregnancy and the postpartum period, new-onset or severe headache necessitates a high index of suspicion for pathology such as pre-eclampsia or eclampsia, which significantly enhance the risk of stroke (Hung et al., 2022; Adekomi et al., 2019). Furthermore, conditions such as RCVS, CVT, and CeAD are important causes of stroke in this demographic that frequently present with unremitting or thunderclap headache (Hadhiah et al., 2021; Carlson et al., 2023; Abdelnour et al., 2022).

While pediatric stroke is less common, headache is a frequent symptom in specific underlying conditions such as systemic vasculitides and Lyme neuroborreliosis; notably in the latter, chronic headache may specifically precede ischemic infarction (Held et al., 2023; Wilke et al., 2000). Other notable pediatric syndromes include RCVS and homozygous sickle cell disease, where vaso-occlusive crises manifest with severe headache and may be complicated by cerebrovascular events (Coffino and Fryer, 2017; Alli et al., 2007).

Beyond these population-specific risks, systemic infections can trigger inflammatory and prothrombotic cascades that lead to both headache and stroke. The COVID-19 pandemic provides a powerful illustration of this link, where patients frequently develop headaches alongside cerebrovascular complications driven by cytokine-mediated endothelial dysfunction (Zhou et al., 2020; Sampaio Rocha-Filho et al., 2022; Meaney et al., 2022). A similar paradigm is observed in tuberculous meningitis, where headache is a cardinal symptom of the infectious process that leads to stroke in approximately 30% of cases (Kumar et al., 2022; Sy et al., 2022).

## 10 Limitations

This review is subject to several limitations inherent to both the review process and the available literature. Primarily, the current evidence base for post-stroke headache relies heavily on retrospective analyses and observational cohort studies, with a notable paucity of randomized controlled trials. This limits the ability to establish definitive causal links or robust treatment guidelines. Furthermore, there is significant heterogeneity across studies regarding the definition and classification of post-stroke headache; the inconsistent application of International Classification of Headache Disorders (ICHD) criteria complicates the stratification of headache phenotypes (e.g., tension-type vs. migraine-like). Additionally, outcome measures vary widely—ranging from subjective pain scales to functional quality-of-life metrics—making direct comparisons between therapeutic interventions difficult. Finally, as a narrative review, this work provides a qualitative synthesis of the literature and may be susceptible to selection bias compared with a systematic review and meta-analysis.

## 11 Conclusion and future directions

Once an overlooked and underdiagnosed symptom, PSH—and particularly PPSH—has had its status as a distinct clinical entity solidified by its formal inclusion in the ICHD-3. Epidemiological data reveal a broad prevalence, affecting a substantial portion of stroke survivors, with rates of new-onset headache ranging from 6 to 44% and persistent headache affecting up to 23% (Harriott et al., 2020; Lai et al., 2018). Well-defined risk factors have been identified, including younger age, female sex, a pre-existing headache history, and stroke location within the posterior circulation or midbrain (Xie et al., 2022; Lai et al., 2018). Furthermore, headache serves as a critical sentinel symptom for a variety of specific and often life-threatening stroke etiologies, such as CeAD, RCVS, and CVT, underscoring the need for a thorough diagnostic workup in patients presenting with acute headache and neurological symptoms (Tabatabai and Swadron, 2016). The impact on patients is clinically relevant, as PSH contributes to comorbid depression, cognitive dysfunction, sleep disturbances, and an overall reduction in quality of life, often acting as a major barrier to successful rehabilitation (Tang et al., 2025).

Despite growing recognition, our understanding of PSH remains incomplete, with significant gaps in knowledge concerning both its underlying mechanisms and its optimal management. The pathophysiology is hypothesized to be multifactorial, involving the activation of the trigeminovascular system, central sensitization, direct damage to central pain pathways, and inflammatory processes, but the precise interplay of these factors and what drives the transition from acute to chronic headache is poorly understood. While we can correlate headache risk with stroke topography, the molecular and cellular mechanisms responsible for this link require further investigation.

A notable gap, however, exists in the realm of treatment. There is a lack of evidence-based guidelines for the acute or preventive treatment of PPSH. Current management strategies are largely extrapolated from treatments for primary headache disorders, a practice that has not been validated in the post-stroke population (Plecash et al., 2019). The safety and efficacy of common analgesics and preventive medications are unknown in these patients. This is particularly true for modern migraine-specific therapies like triptans and CGRP antagonists, whose vascular mechanisms raise theoretical safety concerns that preclude their use without dedicated research. This leaves clinicians and patients with limited, unproven options for a condition that carries a significant disability burden.

The identified knowledge deficits highlight the need for future research. To advance the field of post-stroke pain, a concerted effort is needed to design and execute high-quality, methodologically sound studies. Prospective, longitudinal studies are warranted to better define the natural history of PPSH, clarify its true prevalence using standardized criteria, and identify robust predictors for its development and chronification. Further investigation into its pathophysiology, using advanced neuroimaging and biomarker analysis, is essential to uncover potential therapeutic targets (Weiller et al., 2010).

Furthermore, RCTs are needed to establish the safety and efficacy of treatments for both acute and PPSH. These trials should evaluate a range of interventions, from simple analgesics and established preventive medications to newer therapies like CGRP antagonists. Non-pharmacological approaches, including neurostimulation, nerve blocks, cognitive behavioral therapy, and rehabilitation strategies, also warrant rigorous investigation (Kubik and Biedroń, 2013). The goal of the research should be the development of the first evidence-based guidelines for the management of PSH. Such guidelines would provide clinicians with the tools needed to confidently and effectively treat this condition, moving practice from empiricism to evidence.

Improving recognition is the first step toward better management. While awaiting evidence from RCTs, a pragmatic, multimodal approach to treatment is reasonable, tailored to the individual’s headache phenotype and comorbidities. This may involve a combination of carefully selected pharmacotherapy, physical rehabilitation to address musculoskeletal contributors, and management of comorbid conditions like depression and insomnia. By better recognizing and managing PSH, healthcare providers can alleviate a significant source of suffering, reduce disability, and facilitate more effective participation in rehabilitation. Ultimately, addressing this common complication is essential for the holistic care of stroke survivors and is a crucial step toward the goal of optimizing their functional recovery and long-term quality of life.

## Glossary

CADASIL: Cerebral autosomal dominant arteriopathy with subcortical infarcts and leukoencephalopathy
CeAD: Cervical artery dissection
CGRP: Calcitonin gene-related peptide
CNS: Central nervous system
COVID-19: Coronavirus disease 2019
CPSP: Central post-stroke pain
CT: Computed tomography
CVT: Cerebral venous thrombosis
ICHD-3: International Classification of Headache Disorders, 3rd edition
ICP: Intracranial pressure
LP: Lumbar puncture
MELAS: Mitochondrial myopathy, encephalopathy, lactic acidosis and stroke-like episodes
MRI: Magnetic resonance imaging
MOH: Medication-overuse headache
RCTs: Randomized controlled trials
RCVS: Reversible cerebral vasoconstriction syndrome
PPSH: Persistent post-stroke headache
PSH: Post-stroke headache
SAH: Subarachnoid hemorrhage
TIA: Transient ischemic attack
VAD: Vertebral artery dissection
